# Delirium Tremens: A Review of Clinical Studies

**DOI:** 10.7759/cureus.57601

**Published:** 2024-04-04

**Authors:** Alan D Kaye, Amanda N Staser, Tiombee S Mccollins, Jackson Zheng, Fouad A Berry, Caroline R Burroughs, Michael Heisler, Aya Mouhaffel, Shahab Ahmadzadeh, Adam M Kaye, Sahar Shekoohi, Giustino Varrassi

**Affiliations:** 1 Department of Anesthesiology, Louisiana State University Health Sciences Center, Shreveport, USA; 2 Department of Medicine, Ross University School of Medicine, Miramar, USA; 3 School of Medicine, American University of the Caribbean, Miramar, USA; 4 School of Medicine, Louisiana State University Health Sciences Center, Shreveport, USA; 5 Department of Pharmacy Practice, Thomas J. Long School of Pharmacy and Health Sciences University of the Pacific, Stockton, USA; 6 Department of Pain Medicine, Paolo Procacci Foundation, Rome, ITA

**Keywords:** alcohol withdrawal syndrome, alcohol withdrawal delirium, dts, dt, delirium tremens

## Abstract

Delirium tremens (DT) is a severe condition resulting from alcohol withdrawal. This review highlights the challenges in diagnosing and managing DT and emphasizes the importance of early recognition and intervention to prevent complications and ensure optimal patient outcomes. The discussion of the pathophysiology of DT, focusing on the neurochemical imbalances involving the neurotransmitters gamma-aminobutyric acid and glutamate, explains how chronic alcohol dependence leads to these imbalances and contributes to the hyperexcitability seen in DT. The management of DT involves ensuring patient safety and alleviating symptoms, primarily through pharmacological approaches, such as benzodiazepines. Closely monitoring vital signs and electrolyte imbalances is necessary due to autonomic dysregulation associated with DT. The mention of the potential complexity of DT when coexisting with other conditions emphasizes the need for additional research to advance comprehension, identify predictive factors, and enhance its management.

## Introduction and background

Delirium tremens (DT) is a serious medical problem that poses significant challenges in diagnosis and management. As the most severe form of alcohol withdrawal syndrome (AWS), DT can lead to life-threatening complications if not promptly recognized and appropriately treated. DT typically occurs in individuals with a history of chronic, heavy alcohol consumption who suddenly reduce or cease their alcohol intake [1-3].

Heavy alcohol use is often defined as consuming five or more drinks on any day or 15 or more per week for men, and four or more drinks on any day or eight or more drinks per week for women, based on the National Institute on Alcohol Abuse and Alcoholism (NIAAA) [4]. It manifests as a constellation of symptoms, including profound confusion, disorientation, hallucinations, tremors, agitation, and autonomic instability [3]. 

The onset of DT widely varies but can occur as early as 48 hours after abrupt alcohol cessation. Furthermore, the clinical status can be complicated by the presence of additional comorbidities, such as hepatic encephalopathy (HE) or other substance use disorders, which may mimic or exacerbate DT symptoms. HE can be differentiated by clinical presentations such as flapping tremors, hallucinations of flashing lights, and slow-slurred speech [2,5,6]. Additionally, symptoms of HE are typically accompanied by signs of liver dysfunction, such as jaundice and ascites. Early recognition and intervention are essential to avoid complications and ensure optimal patient outcomes [5].

The pathophysiology of DT involves a cascade of neurochemical imbalances resulting from sudden alcohol withdrawal. Chronic alcohol dependence in individuals develops adaptations in the central nervous system (CNS), leading to imbalances of the neurotransmitters gamma-aminobutyric acid (GABA) and glutamate [1-3,5]. Alcohol is a neurological depressant that exerts its effects by downregulating GABA receptors and upregulating the expression of N-methyl-D-aspartate (NMDA) receptors, leading to an increase in glutamate in the CNS. The imbalance between GABAergic inhibition and glutamatergic excitation contributes to the hyperexcitability seen in DT. The excessive glutamate release and subsequent overactivation of NMDA receptors can lead to excitotoxicity, causing neuronal damage, and further exacerbating the symptoms of DT, such as confusion, hallucinations, and tremors [5].

The management of DT revolves around two primary goals: (1) ensuring patient safety and (2) alleviating symptoms. Early identification and prompt initiation of appropriate interventions are vital for preventing complications and reducing mortality rates [1,7]. Pharmacological approaches, including benzodiazepines and other supportive medications, play a central role in managing the acute manifestations of DT [2,3,5,7]. Close monitoring of vital signs, hydration status, and electrolyte imbalances is imperative, as DT can be associated with significant autonomic dysregulation [5].

DT represents a critical and challenging aspect of AWS, necessitating comprehensive research efforts. By elucidating the underlying mechanisms, identifying predictive factors, and refining management strategies, we can strive toward improved outcomes for individuals affected by DT [8]. This review aims to explain the current understanding of DT, considering recent advancements in research and clinical practice. We hope that through this comprehensive analysis of potential risk factors, clinical presentation, and management of DT, we can enhance the knowledge of healthcare professionals and improve patient care and outcomes.

## Review

Clinical presentation of DT

A combination of delirium and alcohol withdrawal symptoms characterizes DT [7,9]. Per the diagnostic and statistical manual of mental disorders, delirium is characterized by a rapid onset of attention and awareness disturbances, including decreased ability to direct, focus, sustain, and shift attention and reduced orientation to the environment. These symptoms represent a marked change from the individual's usual level of functioning and fluctuate in severity throughout the day. Additionally, there may be disturbances in cognition, such as memory deficits, disorientation, language difficulties, impaired visuospatial ability, or altered perception. Evidence from the individual's history, physical examination, or laboratory findings typically indicates the presence of another medical condition, substance intoxication or withdrawal, toxin exposure, or multiple causes [9-11].

Two main criteria characterize alcohol withdrawal. First, there must be a cessation or reduction in heavy and prolonged alcohol use. Second, two or more of the following symptoms must develop within several hours to a few days after the reduction in alcohol use: autonomic hyperactivity (such as sweating or a pulse rate greater than 100 bpm), increased hand tremors, insomnia, nausea or vomiting, transient visual, tactile, auditory hallucinations or illusions, psychomotor agitation, anxiety, or generalized tonic-clonic seizures. Additionally, these symptoms can impair social and occupational functions [9,12]. DT typically occurs 48-72 hours after the last drink and lasts approximately one to eight days [7,9,13].

The Clinical Institute Withdrawal Assessment of Alcohol Scale (CIWA-Ar) is frequently employed to gauge the severity of alcohol withdrawal and anticipate the onset of DT, as depicted in Table 1. In the CIWA-Ar scale, each item is scored from 0 to 7, with 0 indicating no symptoms, 1-3 indicating mild symptoms, and 4-7 indicating moderate to severe symptoms. A total score above 15 during alcohol withdrawal, particularly in patients with a systolic blood pressure greater than 150 mmHg or a pulse rate greater than 100 beats per minute, may indicate an increased risk of delirium [7,14].

**Table 1:** CIWA-Ar criteria Reference: [14] CIWA-Ar: Clinical Institute Withdrawal Assessment of Alcohol Scale

Symptom	Description	Score
Agitation	Excessive motor activity, restlessness, or irritability	0-7
Anxiety	Subjective distress, nervousness, or a feeling of unease	0-7
Auditory disturbances	Abnormal auditory experiences such as hearing sounds that are not present	0-7
Headache	Presence of a headache or pain in the head	0-7
Nausea/vomiting	Presence of nausea or vomiting symptoms	0-7
Orientation and clouding of sensorium	Level of consciousness, orientation to time and place, and clarity of thinking	0-4
Paroxysmal sweats	Sudden onset of sweating, not explained by external factors	0-7
Tactile disturbances	Sensory disturbances such as itching, tingling, or numbness	0-7
Tremor	Observable tremor of the outstretched hands	0-7
Visual disturbances	Visual hallucinations or other disturbances in vision	0-7
Total	Sum of scores for all criteria	0-67

DT may be obscured by other medical conditions, highlighting the need to consider co-occurring factors. These can include infections, abnormalities in blood chemistries like hyponatremia (which can present as hypoactive delirium), and Wernicke syndrome [5]. These complexities can make diagnosing DT more challenging, potentially leading to delayed treatment and worse outcomes [15]. Wernicke syndrome, caused by thiamine deficiency and often occurring during alcohol withdrawal, can contribute to the development of delirium [15,16]. Additionally, hallucinations, perceptual disturbances, anxiety, and seizures may also co-occur with alcohol withdrawal and withdrawal delirium. The kindling hypothesis and clinical evidence suggest a correlation between the number of alcohol detoxifications and the risks of alcohol withdrawal complications, like seizures, increases due to the cumulative increases in brain excitability [15,17].

Case studies have demonstrated instances of prolonged DT despite intensive medical management. In one case involving a 35-year-old man, DT persisted for 35 days despite high doses of diazepam and haloperidol [13]. Another case involved a 48-year-old male with chronic alcohol dependence, whose symptoms worsened despite initial lorazepam tapering and ICU admission for DT. Despite ruling out other causes and escalating benzodiazepine therapy, symptoms persisted until a combination of midazolam infusion and phenobarbitone led to successful symptom control over 28 days [18]. Both cases highlight the challenges and variability in managing prolonged DT, with successful outcomes achieved through individualized treatment approaches [5].

Pathophysiology and neurobiology of DT

GABA is a signaling messenger that alters chemicals in the brain to significantly reduce the neural excitability in the CNS. Like many other neurotransmitters, GABA sends messages by binding to their respective receptors in the synapse between nerve cells. The GABA neurotransmitter, the most common neurotransmitter known for its inhibitory mechanism, has two target receptors on the post-synaptic cell: the GABAA receptor and the GABAB receptor [19]. The GABAA receptor is a ligand-gated chloride channel that subsequently increases the movement of negatively charged ions, such as chloride, to the nerve cell. As negatively charged chloride ions move into the nerve cell, inhibitory post-synaptic potentials (IPSPs) are produced, which makes a post-synaptic neuron unable to create an action potential properly [20]. The GABAB receptor is a G protein-coupled receptor that increases positively charged potassium conductance and decreases positively charged voltage-gated calcium ion channels, which will suppress adenylate cyclase and produce an IPSP, ultimately leading to a neural inhibitory action [21].

In contrast, glutamate is the primary excitatory neurotransmitter for spinal cord and brain nerve cells. Glutamate, like GABA, is a signaling messenger that sends signals between nerve cells by attaching to their respective glutamate receptors in the synaptic cleft of the post-synaptic cell [22]. However, unlike GABA, glutamate will excite and activate a nerve cell that will induce an excitatory post-synaptic potential and propagate a chain reaction. Glutamate is constantly salvaged and re-processed by neural glial cells to be converted into glutamine, which can then be transformed into glutamate or GABA [23].

In excessive alcohol consumption, alcohol significantly activates GABA receptors, encouraging inhibition of neuronal activity [24,25]. As more alcohol is consumed excessively, tolerance begins to build so that more alcohol is required to sustain the inhibition effect of GABA. With increased alcohol consumption, glutamate is produced in higher amounts to compensate for the inhibitory effects. Once alcohol is removed and undergoing withdrawal, the GABA inhibitory stimulation ceases. However, excess glutamate affects the NMDA receptors by overloading the nerve cells with excitatory neurotransmitter function [26]. This increases susceptibility to neuronal cell death due to excitotoxicity [27]. The clinical signs and symptoms manifest in palpitations, anxiety, tremors, headaches, restlessness, and seizures [28].

The effects of withdrawal from alcohol may surface within a few hours of halting alcohol consumption, depending on the history of alcohol usage. In patients with a known history of significant alcohol consumption, an immediate cessation could result in a tonic-clonic seizure or convulsion. In delirium, tremens, hallucinations, agitation, extreme sweating, and altered mental status can also occur. In addition to hypophosphatemia and hypomagnesemia, hypokalemia during alcohol withdrawal may occur due to the loss of potassium via inappropriate kaliuresis and an increase in aldosterone levels [28-30].

Management of DT

Management of DT includes pharmacological and non-pharmacological approaches. The initial assessment for DT typically begins with preliminary laboratory studies, including a complete metabolic panel, complete blood count, and liver function tests such as prothrombin time [24]. However, the specific tests conducted may vary depending on the patient's presentation. Other studies that may be considered during the assessment of DT include imaging studies such as CT scans or MRIs of the brain to rule out other causes of altered mental status, as well as toxicology screens to identify any coingestants or substances contributing to the delirium. Additionally, assessments of vital signs, including blood pressure, heart rate, respiratory rate, and temperature, are crucial in monitoring the patient's condition and guiding treatment [24]. Treating underlying medical problems that may have contributed to the acute state is critical. The chief goal of treatment in delirium withdrawal is seizure prevention, control of patient distress, and decreased risk of injury or death [8]. Due to the increased lethal risk, treatment demands an inpatient psychiatry setting or ICU unit to monitor vitals and laboratory parameters [7].

Treatment begins by establishing a functioning intravenous line. The basis of a pharmacological approach most commonly utilizes depressants such as benzodiazepines [31]. Benzodiazepines have a mechanism of action that allosterically binds to the GABAA receptors. This leads to an increased influx of chloride and the hyperpolarization of the neuron's action potential. This increased frequency of the chloride channel opening results in CNS downregulation [32-34]. All drugs of this class appear to have the same efficacy [35]. Conversely, the probability for misuse is greater with benzodiazepines that have a rapid onset of action, including diazepam and lorazepam, than for those with a slower onset of action, such as oxazepam or chlordiazepoxide [32]. The quantity needed to regulate insomnia and agitation varies considerably among patients (e.g., >2000 mg of diazepam in the first two days in some patients) [7]. An 11-trial meta-analysis with 1286 participants compared patients taking benzodiazepines with an active control drug or placebo. A clinically significant decline of symptoms was seen within two days with benzodiazepines than with a placebo or a control drug [36].

Adjuvant treatments include propofol, dexmedetomidine, phenobarbital, haloperidol, or carbamazepine [37]. For patients who do not respond to high doses of benzodiazepines, up to 4 mg per kilogram per hour for up to 48 hours of propofol may be administered. This option is best for patients who are intubated. Dexmedetomidine, an α2-adrenergic agonist, can be administered in doses up to 0.7 μg per kilogram per hour. It creates a state in which the patient is sedated but still arousable, with a reduced sympathetic tone. While there are no definitive contraindications for using dexmedetomidine, it is advised to exercise caution in vulnerable patient groups, such as those with left ventricular dysfunction or heart failure, as there is some evidence suggesting that it could potentially worsen cardiac function [38]. Barbiturates, such as phenobarbital, have been shown to provide improvement in alcohol withdrawal. A potential side effect is respiratory depression when used in high doses. Haloperidol, an antipsychotic drug, is used for severe hallucinations or agitation. Antipsychotic medications can prolong the QT interval and increase the probability of seizure occurrence. Anticonvulsant agents, such as carbamazepine, are given at an initial dose of 800 mg daily. These drugs are equally effective compared to oxazepam and phenobarbital in treating mild-to-moderate withdrawal symptoms. Carbamazepine has no potential for abuse or toxic effects on the liver when used in a seven-day protocol [36].

Early supplement with thiamine (vitamin B1) is essential. High doses (300 mg infused intravenously over 30 minutes once or twice daily for three days) of thiamine should be administered before glucose in DT. If a patient is suspected of having Wernicke's encephalopathy, a higher dose of thiamine (e.g., 500 mg intravenously three times daily for five days) is recommended. Exercise caution when administering pure glucose to prevent the risk of triggering Wernicke's encephalopathy or cardiomyopathy. These complications can develop quickly due to consuming the last thiamine reserves in glycolysis. Treatment should be continued for 7-14 days. Premature medication discontinuation could result in delirium relapse within 24 hours [7].

Non-pharmacological approaches in DT management include the same standard methods of support required for all patients with delirium. These include adequate hydration, frequent checking of vital signs, treating the patient in a well-lit, quiet room, reassuring, and reorienting the patient to date, time, and place [5,7]. Incorporating motivational interviewing techniques into the earliest phases of community-based outpatient treatment, such as the evaluation session, has been suggested to affect patient retention positively [39]. After delirium subsides, education and supportive therapy for patients is vital. This therapy aims to prevent shame or guilt, ensure the patient's understanding of DT symptoms, and aid with reintegration into their original environment. These steps may have a pronounced impact on the patient's motivation to initiate the long-term treatment of alcohol dependence [28]. Globally, 2.8 million deaths were attributed to alcohol use in 2016. Alcohol was the primary risk factor for disability and premature death for individuals aged 15-49. Findings indicate that males with alcohol use acquire far more health loss than females [40]. Mortality rates due to DT are as great as 20%. Early identification and appropriate treatment decrease the rate to 1%. A previous withdrawal delirium episode seems to predict recurrent future development [28].

Discussion

DT is a severe and complex medical condition arising from alcohol withdrawal. It poses significant challenges in diagnosis and management due to its potentially life-threatening complications if not promptly recognized and appropriately treated. Symptoms, including profound confusion, disorientation, hallucinations, tremors, agitation, and autonomic instability, characterize DT. Based on the presentation of symptoms, treatment should be targeted toward the possible dysregulation of GABA and glutamate within the CNS. DT typically occurs in individuals with a history of chronic, heavy alcohol consumption who suddenly reduce or cease their alcohol intake. Comprehensive research efforts are needed to improve outcomes for individuals affected by DT. We can strive toward improved patient care and outcomes by elucidating the underlying mechanisms, identifying predictive factors, and refining management strategies. Research should focus on understanding the neurobiology of DT, identifying risk factors, and developing targeted interventions. Furthermore, addressing alcohol use as a public health issue is paramount. Alcohol-related harm, including DT, contributes to significant morbidity and mortality rates worldwide. Table 2 provides an overview of the clinical findings and the prevalence of DT in various populations. These studies helped identify high-risk patients who developed DT. The demographic profile of these populations typically consisted of middle-aged males with comorbidities and a history of long-term alcohol abuse [40-46]. Efforts should be directed toward prevention, early intervention, and long-term treatment of alcohol dependence to reduce the burden of DT and associated complications. A holistic understanding of the etiology of DT, including sociological disparities, can lead to more effective prevention and treatment strategies [42].

**Table 2:** Comparative studies of delirium tremens DT: delirium tremens; AOR: odds ratio for age

	Author (Year)		Type of Study	Country	Population Studied	Results of Those Who Developed DT	Risk Factors
Study 1	Pribék (2023) [41]		Retrospective study	Hungary	1,591 patients and 2,900 hospital visits	Incidence: 36.8%	Higher mean age, comorbid somatic disorders, and lower temperatures in March
Study 2	Joshi (2021) [42]		Cross-sectional study	Nepal	105 patients with alcohol withdrawal syndrome	Prevalence: 69.52%	Middle-aged, illiterate, manual laborers. Consumptions of homemade alcohol and medical comorbidities
Study 3	Paudyal (2020) [43]		Case-control study	Nepal	Alcohol-dependent patients	24% patients developed DT odds ratio: age >50 years old, AOR=75.7, 95% (CI=7.8-730); alcohol consumption >20 years, AOR=305, 95% (CI=3.4-2711); 48-72 hours since the last intake of alcohol AOR=923, 95% (CI=38-22333)	Older age, longer duration of alcohol intake, and longer time interval since last alcoholic drink
Study 4	Teetharatkul (2018) [44]		Cross-sectional study	Thailand	Alcohol-dependent patients	Prevalence: 28.0%	Red blood cell count <4.5x106/ul (p-value<0.001)
Study 5	Moore (2017) [45]		Retrospective study	United States of America	345,297 veterans with alcohol use disorder (AUD)	Incidence: 0.7%	Homelessness, comorbid disorders, health service utilization

## Conclusions

To summarize, DT represents a severe form of AWS, marked by profound confusion, hallucinations, tremors, and autonomic instability. Early recognition and intervention are paramount to prevent complications and ensure optimal patient outcomes. This review underscores the significance of comprehending DT's pathophysiology, clinical presentation, and management strategies, emphasizing the necessity for ongoing research and public health initiatives to combat alcohol-related harm and enhance patient care.
